# Interactive virtual 3D models of renal cancer patient anatomies alter partial nephrectomy surgical planning decisions and increase surgeon confidence compared to volume-rendered images

**DOI:** 10.1007/s11548-019-01913-5

**Published:** 2019-01-24

**Authors:** E. R. Hyde, L. U. Berger, N. Ramachandran, A. Hughes-Hallett, N. P. Pavithran, M. G. B. Tran, S. Ourselin, A. Bex, F. H. Mumtaz

**Affiliations:** 1 School of Biomedical Engineering and Imaging Sciences, King’s College London, London, UK; 2 Innersight Labs Ltd, London, UK; 3 Department of Radiology, UCLH NHS Foundation Trust, London, UK; 4 Specialist Centre for Kidney Cancer, Department of Urology, The Royal Free London NHS Foundation Trust, London, UK; 5 University College London Division of Surgery and Interventional Science, London, UK

**Keywords:** Renal masses, Urological oncology, Computed tomography, Interactive virtual 3D model, Surgical planning

## Abstract

**Purpose:**

To determine whether the interactive visualisation of patient-specific virtual 3D models of the renal anatomy influences the pre-operative decision-making process of urological surgeons for complex renal cancer operations.

**Methods:**

Five historic renal cancer patient pre-operative computed tomography (CT) datasets were retrospectively selected based on RENAL nephrectomy score and variety of anatomy. Interactive virtual 3D models were generated for each dataset using image segmentation software and were made available for online visualisation and manipulation. Consultant urologists were invited to participate in the survey which consisted of CT and volume-rendered images (VRI) for the control arm, and CT with segmentation overlay and the virtual 3D model for the intervention arm. A questionnaire regarding anatomical structures, surgical approach, and confidence was administered.

**Results:**

Twenty-five participants were recruited (54% response rate), with 19/25 having > 5 years of renal surgery experience. The median anatomical clarity score increased from 3 for the control to 5 for the intervention arm. A change in planned surgical approach was reported in 19% of cases. Virtual 3D models increased surgeon confidence in the surgical decisions in 4/5 patient datasets. There was a statistically significant improvement in surgeon opinion of the potential utility for decision-making purposes of virtual 3D models as compared to VRI at the multidisciplinary team meeting, theatre planning, and intra-operative stages.

**Conclusion:**

The use of pre-operative interactive virtual 3D models for surgery planning influences surgical decision-making. Further studies are needed to investigate if the use of these models changes renal cancer surgery outcomes.

**Electronic supplementary material:**

The online version of this article (10.1007/s11548-019-01913-5) contains supplementary material, which is available to authorized users.

## Introduction

The evolution and uptake of laparoscopic and robotic-assisted techniques have led to an increase in the use of minimally invasive partial nephrectomy (PN), also known as nephron-sparing surgery, for the treatment of the small renal mass [1–3]. A key driver behind this trend is the accumulating evidence that post-operative healthy kidney volume is positively correlated with improved kidney function as measured by the estimated glomerular filtration rate (eGFR) [4, 5]. Surgical planning for minimally invasive PN is complex, with numerous patient and tumour characteristics having to be accounted for, especially the relationship between the tumour and renal hilar anatomy. Historically, the appreciation of these anatomical factors has been through the examination of coronal, sagittal, and axial slices of computed tomography (CT) and magnetic resonance imaging (MRI) datasets. Recently, the use of volume-rendered images (VRI) for surgery planning has become more common [6], but VRI has significant limitations. VRI can only reveal structural information when tissue contrast is high, VRI can only be applied to a single scan at a time, and VRI supplies no semantic knowledge. Therefore, quantitative analysis such as distance/area/volume measurements cannot be undertaken and typically the relationship of the tumour to collecting or venous systems cannot be appreciated when using VRI.

To overcome the general limitations of VRI in a soft tissue oncology setting, dedicated software packages have been developed to classify medical scan voxels into their anatomical components in a process known as *image segmentation* [7–9]. Once segmented, stereolithography files are generated which can be used to visualise the anatomy and have the components 3D printed. It has been shown that such 3D printed models influence surgical decision-making and patient education [10, 11]. However, the relevance of a physical model to plan for a minimally invasive surgical approach is debatable, and the financial and administrative costs of obtaining accurate 3D printed models for routine surgery planning have been speculated to be holding back 3D printed models from breaking into regular clinical usage [12].

As a necessary precursor to 3D printed models, computational or virtual 3D models could be used by the urologist to assist with clinical decision-making. Virtual 3D models provide many of the advantages of their physical 3D printed counterpart without the challenge of the printing process. They can be easily viewed on standard digital devices such as laptops or smartphones, and they can be simultaneously viewed from anywhere in the world which could help with collaborative surgery planning between centres. Furthermore, the ability of the viewer to interact with the virtual 3D models, e.g. selecting which anatomical components are visible or setting preferred viewpoints, intuitively should lead to efficiencies in learning the important anatomical features for the given clinical application. For example, pioneering studies have already shown that surgeons benefit from virtual 3D models in the theatre, particularly with regard to improved appreciation of hilar vascular anatomy and noteworthy attempts to minimise ischaemic period and volume through enhanced pre-operative planning and simulation [13, 14]. However, in addition to the available routine imaging (CT, MRI, VRI), it has not been shown that virtual 3D models would influence the surgical decision-making process or alter surgeon confidence in their decisions.

The objective of the current study is to determine whether the interactive visualisation of patient-specific virtual 3D models of the renal anatomy influences the pre-operative decision-making process of urological surgeons for complex renal cancer operations. A secondary objective is to determine whether or not surgeon confidence levels can be increased by the addition of virtual 3D models, which could have a positive impact on surgeon stress levels [15].

## Methods

### Imaging data

**Fig. 1:**
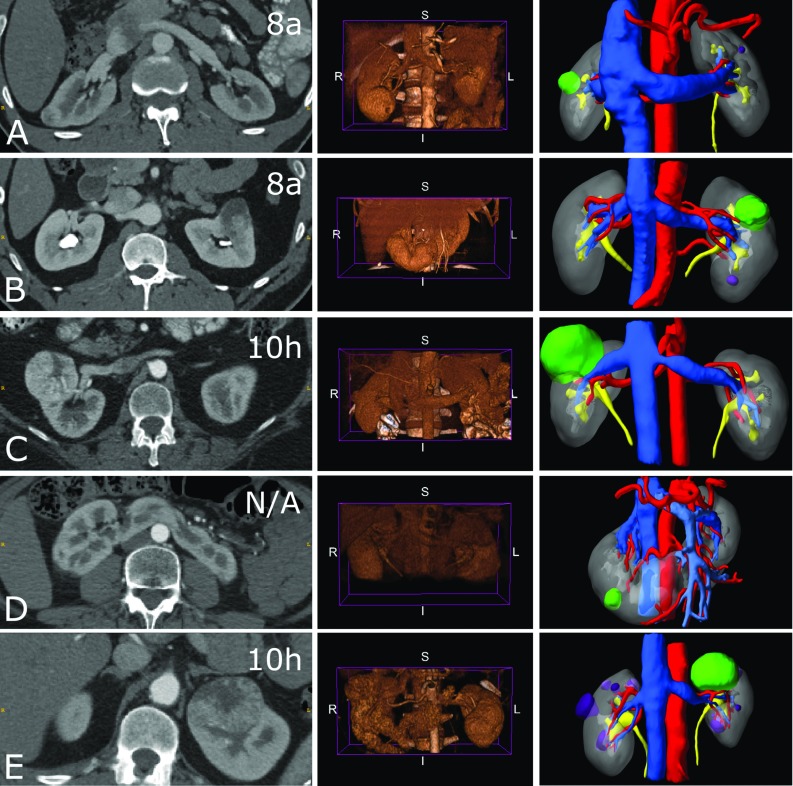
Representative imaging available for the 5 study cases **a**–**e** (rows) featuring: (left) arterial phase CT axial slice with the case RENAL score indicated in the top right corner. The horseshoe kidney case has not been given a RENAL score. (Middle) Arterial phase abdominal CT volume-rendered images (VRI) from an anterior viewpoint. (Right) A static screenshot of the generated, case-specific, interactive virtual 3D model which featured in the intervention arm of this study. The structure—colour keys are: artery—red; venous—dark blue; portal venous—light blue; tumour—green; cyst—purple; excretory—yellow; normal kidney—grey

A registry of patients who received clinically indicated and standard-of-care CT prior to surgery for renal masses between 2015 and 2017 was retrospectively reviewed by a urologist with expertise in kidney cancer surgery to identify 5 cases with RENAL nephrometry score greater than 7 (Table 1; Fig. 1, left). This study was approved by the local institutional review board at the Royal Free London NHS Foundation Trust, University College, London.

**Table 1:** Descriptive features of the five renal cancer cases selected for the survey

Case	Tumour location	Tumour diameter (mm)	RENAL score	PADUA score	Comments
A	Ant, Int, Lat	21	8a (1, 3, 1, a, 3)	9 (1, 3, 2, 1, 1, 1)	
B	Ant, Int, Med	31	8a (1, 2, 3, a, 2)	11 (1, 3, 2, 2, 1, 2)	Multiple renal arteries
C	Ant, Int, Hil	54	10 h (2, 2, 3, h, 3)	12 (2, 2, 2, 2, 2, 2)	
D	Pos, Low/Int, Lat	18	N.A. ()	N.A. ()	Horseshoe with portal vein
E	Ant, Upp/Int, Med/Hilar	57	10 h (2, 2, 3, h, 3)	12 (2, 2, 2, 2, 2, 2)	

Nephrectomy scores are followed by their component-wise breakdown. Letters A–E correspond to the cases A–E illustrated in Fig. 1

*Ant* anterior, *Pos* posterior, *Upp* upper pole, *Int* interpole, *Low* lower pole, *Lat* lateral, *Med* medial, *Hil* hilar

The study intervention arm consisted of the CT scans including renal arterial, venous and excretory phases, and a patient-specific interactive virtual 3D model. Patient CT scans were anonymised and imported into a dedicated renal model generation platform (Innersight Labs Ltd, London, UK). This software was used to register intra-patient CT phases and perform the image segmentation task, thereby partitioning the scan voxels into pre-selected classes, namely, background, renal artery, renal venous, renal excretory, normal renal tissue, and abnormal renal tissue. All study models can be viewed from following the publications page links provided at https://innersightlabs.com. The model generation algorithm has previously been described [16], and all image processing was performed by a research scientist with 7 years of medical image processing experience. The output of the software package was a set of Visualization ToolKit (VTK) files detailing the surfaces of all objects of interest for each case where each virtual 3D model could be interacted with including viewing from any angle (Supplementary Figure S1 shows anterior and posterior views for all models), and selective structure visibility and transparency (Fig. 1, right).

The study control arm consisted of the CT scans and a comprehensive set of VRI, which were created using the Ray Cast algorithm within the medical image visualisation software package, *3D Slicer* (Kitware Inc, NY, USA) [8]. Transfer functions were manually tuned by a radiologist to optimise contrast differences between anatomical objects of interest. Two rotational axes were defined around the Inferior–Superior and Left–Right lateral axes, with each full rotation being divided in 10 segments, giving a total of 100 VRI for each available CT phase, all of which were made available for online viewing (Fig. 1, middle).

### Survey recruitment and content

Survey participants were restricted to consultant urological surgeons that conduct a minimum of 10 renal cancer procedures *p.a.* Participants were offered no incentives to reply. The survey was hosted on a secure online platform (www.surveymonkey.com) and consisted of a series of alternating control and intervention arm questionnaires. Participants viewed all datasets, but the order of the intervention arm cases was randomised such that an intervention arm case was never directly preceded or followed by its associated control arm to minimise potential confounding bias. The questionnaire was designed to investigate surgeon opinion on (a) the clarity of the imaging provided; (b) surgical complexity; (c) feasibility of partial nephrectomy; (d) surgical approach (open/laparoscopic/robotic); (e) surgeon confidence; and (f) usefulness of VRI or virtual 3D models in addition to CT images for surgery planning (for the full questionnaire, see Table 2).

**Table 2:** Pre-operative urologist questionnaire regards to available planning aids and their impact on surgical approach

Question	Response options
Q1 [both arms] There is a single tumour in the left/right/horseshoe kidney, please describe its exact location by selecting one option from each drop-down menu	Anterior/posterior Upper-/inter-/lower-pole Lateral/medial/hilar
Q2 [both arms] How clearly does the imaging information provided indicate the relationship of the tumour to the following systems? (Please choose one of 1–5, 1 being “Very unclear” and 5 being “Very clear”)	Arterial system (1–5) Venous system (1–5) Collecting system (1–5)
Q3 [both arms] How would you rate the surgical complexity of this case on visual inspection? (Please choose one of 1–5, 1 being “Simple” and 5 being “Very complex”)	1–5
Q4 [both arms] What is the feasibility of carrying out a partial nephrectomy based on the images provided? (Please choose one of 1–5, 1 being “Not feasible” and 5 being “Very feasible”)	1–5
Q5 [both arms] If you had to choose partial nephrectomy, what would be your preferred approach?	Open/laparoscopic/robotic
Q6 [both arms] If you had to choose partial nephrectomy, and based on the images provided, how confident are you in carrying out segmental vascular clamping? (Please choose one of 1–5, 1 being “Not confident” and 5 being “Very confident”)	1–5
Q7 [control] Does seeing these VR images add any extra useful information to your surgical planning over CT alone? (Please choose one of 1–5, 1 being “No extra useful information” and 5 being “Significant extra information”)	1–5
Q7 [intervention] Does interacting with the 3D model add any extra useful information to your surgical planning over CT alone? (Please choose one of 1–5, 1 being “No extra useful information” and 5 being “Significant extra useful information”)	1–5
Q8 [control] How useful is the provided imaging (CT + VR) for surgical planning? (Please choose one of 1–5, 1 being “Not useful” and 5 being “Very useful”)	MDT (1–5) Theatre planning (1–5) intra-operative (1–5)
Q8 [intervention] How useful is the provided imaging (CT + 3D) for surgical planning? (Please choose one of 1–5, 1 being “Not useful” and 5 being “Very useful”)	MDT (1–5) Theatre planning (1–5) Intra-operative (1–5)

Forty-six eligible surgeons were invited to take the survey, with 25 participants from 21 medical centres and 7 countries (Belgium, Czech Republic, France, Italy, Netherlands, Slovenia, UK) completing the survey (54% response rate; experience level: 1–5 years, *n* = 6; > 5 years, *n* = 19). The average overall number of renal operations conducted per participant per annum was 34.5 ± 25.2 (mn ± SD) with a minimum of 10 and a maximum of 100. The participants had experience in a variety of surgical approaches with 22/25, 21/25, and 17/25 having access to open, laparoscopic, and robotic options, respectively.

### Data analysis

Categorical variables are frequencies (percentages) and median scores. Qualitative scores (i.e. Likert items) are quantified by the nonparametric Wilcoxon signed-rank test with Pratt treatment of zero-differences. The null hypothesis tested is that the distribution of the intervention score minus the control score is symmetric abut zero, i.e. that there is no difference between study arms. A *p* value < 0.05 was considered statistically significant. Statistical analysis was performed using Python’s statistical functions (Python 3.6.0; SciPy v0.19.1).

To define a single measure of total anatomical clarity improvement (∆TAC), the sum of the individual component clarity scores under control imaging is subtracted from the equivalent for the intervention imaging. Specifically, by denoting the anatomical clarity Likert score (AC) for the arterial, venous, and excretory systems with subscripts *a*, *v*, and *x*, respectively, and the control and intervention arms with superscripts *c* and *i*, respectively, we define the total change in anatomical clarity as judged by the user to be

$$ \Delta {\text{TAC}} = \mathop \sum \limits_{{s \in \left\{ {a,v,x} \right\}}} \left( {{\text{AC}}_{s}^{i} - {\text{AC}}_{s}^{c} } \right) $$

Given that each AC value is an integer in the range [1, 5], *∆*TAC must be an integer in the range [− 12, 12]. A positive value of *∆*TAC implies that the participant believed themselves to have gained a better understanding of the overall patient anatomy from the virtual 3D model than from VRI.

## Results

### Anatomical–spatial awareness

With regard to survey question 1, there was no statistical difference in the ability of surgeons to correctly locate the tumour between study arms, with similar ability to do so whether using control imaging (76%) or intervention imaging (78%).

**Fig. 2:**
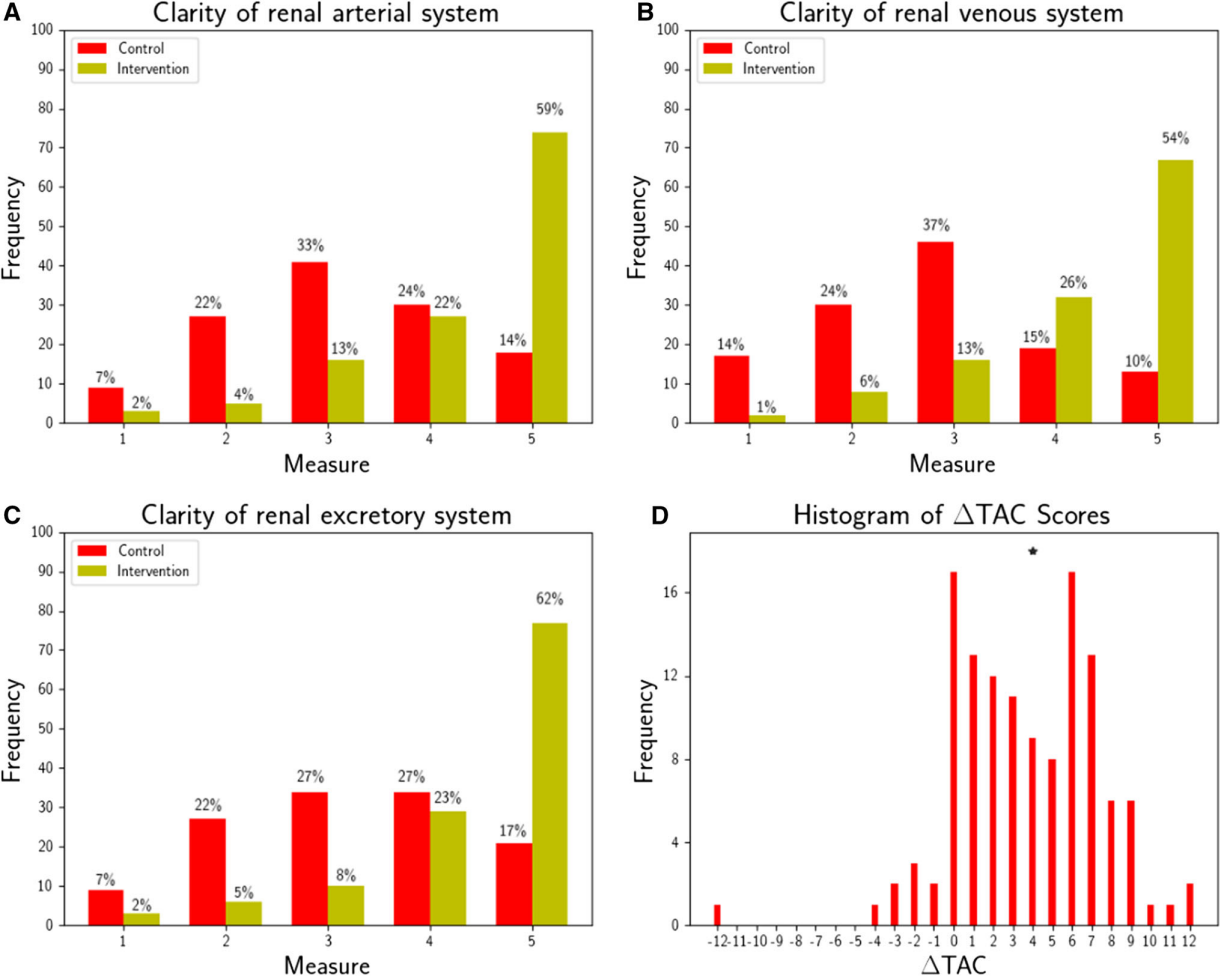
Comparison of the impact of control imaging (CT and volume-rendered images) and intervention imaging (CT and interactive virtual 3D model) on surgeon comprehension of renal anatomy. Clarity of anatomical–spatial location of the arterial (**a**), venous (**b**), and the excretory (**c**) systems was measured using a 5-point Likert scale where 1 was “Very unclear” and 5 was “Very clear”. A statistically significant improvement (*p *< 0.05) in total anatomical clarity was observed (**d**), with a median difference in total anatomical clarity score (*∆*TAC; see “Data analysis” section) of 4 (denoted by the asterisk). If there was no difference in surgeon opinion between the imaging types of each study arm, the distribution of *∆*TAC would be symmetric about zero and have a median value of zero

Virtual 3D models were judged by participants to produce better anatomical clarity than VRI. When asked “On the scale of 1–5, 1 being “Very unclear” to 5 being “Very clear”, how clearly does the imaging information provided indicate the relationship of the tumour to the following systems?” (survey question 2), fewer patient datasets scored in the range 4–5 using control imaging (proportion of scores in the range 4–5 per component system: arterial 38%, venous 25%, excretory 44%) as compared to intervention imaging (proportion of scores in the range 4–5 per component system: arterial 81%, venous 80%, excretory 85%) (Fig. 2a–c). Across the three component systems, the median control arm anatomical clarity score was 3 (“Reasonably clear”), whereas the median intervention arm anatomical clarity score was 5. For each individual component system, there was a statistically significant difference between the perceived anatomical clarity under each study arm (*p* < 0.05). Combining the component anatomical clarity scores into the difference of total anatomical clarity, *∆*TAC, the distribution of this measure is skewed in favour of better anatomical clarity for the intervention imaging with a median *∆*TAC value of 4 (Fig. 2d). This difference was found to be statistically significant (Wilcoxon test statistic 180.0, *p* < 0.05).

### Clinical decision-making and confidence

**Fig. 3:**
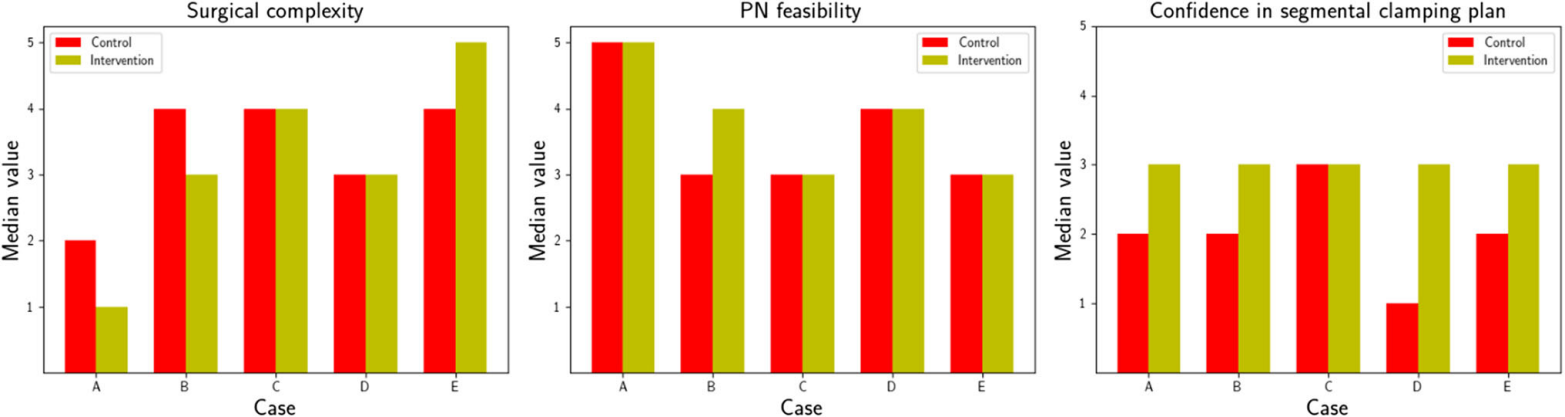
Barcharts of median values per factor per case as judged by the surgeon, assuming that a partial nephrectomy (PN) was to be undertaken: surgical complexity (left), PN feasibility (centre), and confidence in the segmental clamping plan (right). Control-to-intervention differences in median complexity were observed for 3/5 cases (A, B, and E). There was an increase in median PN feasibility for case B only. There was an increase in median clamping strategy confidence in 4/5 cases, including a 2-point increase for the horseshoe kidney dataset, case D

The inter-arm median values of respondent opinion on surgical complexity were different in 3/5 patient datasets (Fig. 3, left; patient datasets A, B & E), with one difference in median PN feasibility (Fig. 3, centre; dataset B), demonstrating the translation of changes in surgeon anatomical–spatial awareness into factors directly related to surgical decisions.

Surgeons changed surgical approach decisions between survey arms for the same patient dataset almost 1-in-5 times (24/125; patient datasets: A × 3, B × 6, C × 5, D × 4, E × 6). Most surgery approach changes occurred from open PN to minimally invasive PN (16/125; patient datasets: B × 4, C × 2, D × 4, E × 6) with fewer approach changes occurring in the reverse manner of minimally invasive PN to open PN (8/125; patient case occurrence breakdown: A × 3, B × 2, C × 3). The median *∆*TAC value for cases that did not involve a surgical approach change from open PN to minimally invasive PN or vice versa was 3, and this value increased to 6 for cases that did experience a change between open and minimally invasive approaches, indicating that major changes in surgical approach were positively correlated with perceived improved anatomical awareness by the surgeon.

Regardless of a change in surgical approach or not, surgeon confidence in their segmental clamping plan increased with interventional imaging for 4/5 cases with a median increase of 1 point on the Likert scale for cases A, B, and E and a median increase of 2 points for case D—the horseshoe case (Fig. 3, right).

### Interactive model utility relative to the patient care pathway

**Fig. 4:**
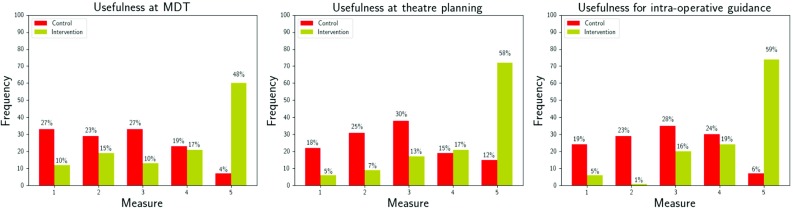
Comparison of urological surgeon opinion on the potential usefulness of control and intervention imaging at three key stages of the renal cancer patient care pathway: the MDT meeting (left), theatre planning (centre), and intra-operative (right). Usefulness of the imaging provided was measured using a 5-point Likert scale where 1 was “Not useful” and 5 was “Very useful”. There was a statistically significant difference in the scores obtained under both study arms across all three stages (*p *< 0.05). *MDT* multidisciplinary team

Surgeons were more positive in their attitude towards interventional imaging rather than control imaging for potential clinical utility at the MDT (*p* < 0.05), theatre planning (*p *< 0.05), and intra-operative (*p *< 0.05) stages of the patient care pathway (Fig. 4). In addition to the survey, the case of the horseshoe kidney (Fig. 1d) is an example of a real change from a planned open to a robotic-assisted partial nephrectomy based on the additional confidence gained from the 3D virtual model.

## Discussion

Pre-operative planning for renal cancer surgery is critical for achieving the best patient outcome. While mindful of key patient co-morbidities, the surgical plan hinges on surgeon awareness of the often complex spatial relationship amongst the various anatomical components. Tumour localisation, nearness to the collecting system, and vascular invasion are all important factors that are not necessarily easy to correctly grasp from the constrained 2D visualisation of CT or MRI data alone. This study aimed to demonstrate a possible improvement in the tools available to the urologist for surgery planning and to investigate how the proposed technology compares to the status quo.

This study is not the first time that virtual 3D anatomical models have been used to aid surgery planning. For example, multiple research groups have previously introduced such models in the Operating Room (OR) via the *da Vinci™* console using the *TilePro™* function (Intuitive Surgical, Sunnyvale, USA) [13, 17]. Although small-scale proof-of-concept studies, they showed that the use of virtual 3D models in the OR did not distract from the procedure and provided useful additional information for surgery guidance purposes. Similar to 3D printed models [18], the results of this study provide further evidence that virtual 3D models when used as an adjunct to surgery planning can improve surgeon understanding of the patient anatomy, as indicated by the significant increase in the surgeon’s opinion of their own anatomical awareness. Arguably the greatest benefit of virtual 3D models is for atypical cases, however, such as high number of renal arteries or horseshoe kidneys [19]. The horseshoe case featured in this study (case D) was the only case to obtain more than a 1-point increase in median surgeon confidence in their segmental clamping plan. It is worth noting that due to a lack of resources or technology not all surgeons have access to volume-rendered images for surgery planning assistance. It could be reasonably assumed that the extra benefit of virtual 3D models from a planning perspective would be even greater than demonstrated in this study when compared to the original multiphase CT scans alone.

This study also investigated the impact of virtual 3D models on the key clinical decision of whether to use an open, laparoscopic, or robotic surgical approach to partial nephrectomy. A change in surgical approach was made in almost 1-in-5 cases (19%) with the only difference between study arms being the form of the imaging information provided. Notably, two-thirds of those approach changes were from an open to a minimally invasive technique, suggesting the potential for treating more patients with nephron-sparing surgery under the interventional imaging protocol. This result provides a clear pathway to impacting patient outcome by using enhanced surgery planning tools.

The clinical utility of virtual 3D models was consistently judged to be significantly greater than that of volume-rendered images (VRI) across the three clinical stages considered: multidisciplinary team meeting, theatre planning, and intra-operative for assisting navigation. Despite the preference for virtual 3D models, the use of such models for surgery planning has still not been translated into common practice. This is likely due to the requirement of significant labour and expertise in manually constructing the virtual 3D models from the original medical scans, as well as the financial cost [20]. This bottleneck of obtaining the pre-operative anatomical segmentation underlies the main challenge to many forms of quantitative surgical assistance, from 3D printing of orthopaedic implant guides [21] to forming the baseline, pre-operative model for augmented reality intra-operative surgical navigation systems [22]. Advances in machine learning techniques, typically using convolutional neural networks (CNNs), have made significant inroads into overcoming this barrier [23, 24]. Our group have recently applied similar CNN methods for fully automated kidney segmentation from contrast-enhanced CT and achieved state-of-the-art Dice scores of over 95% for both the left and right kidneys [16]. Continued improvements in computer vision image segmentation algorithms should enable similarly accurate models to be constructed for all abdominal organs within clinically relevant timeframes, which is already obtained using slower computational methods [25]. Extensions of this work to more challenging aspects such as abnormal tissue classification [26], vessel segmentation [27, 28], and methods of efficiently incorporating user interaction to improve model generation are underway across several research groups [29]. Moreover, scalable virtual 3D model generation could lead to the adoption of improved methods of not just surgery planning but also patient-specific pre-operative training [30].

This study has its limitations. Firstly, it featured analysis from 25 respondents using a qualitative questionnaire. The number of respondents is in line with or larger than other similar studies [10, 13], but extrapolation to the general urologist population should be treated with caution. Secondly, the study’s 54% response rate may also result in selection bias with respondents perhaps being those most amenable and interested in the use of virtual 3D imaging. Thirdly, study data analysis was mostly restricted to simple frequency and median statistics due to the use of qualitative Likert items [31], but statistical power was achieved by considering all 125 surgeon dataset combinations for testing overarching hypotheses not specific to any one patient. Furthermore, the study design of comparing the intervention to control imaging renders analysis of inter-rater agreement to be nonmeaningful. Specifically, the use of virtual 3D models made surgeons generally believe that they had a better spatial awareness of the patient body which resulted in a majority of high Likert scores causing an unbalanced results dataset which is unsuitable to Fleiss Kappa statistical analysis. Fourthly, the virtual 3D models are not yet perfect representations of the patient anatomy. For example, in Fig. 1b (right) the combination of finite spatial resolution of the image and partial volume effects has resulted in two separate, traversing arteries to appear fused. More algorithmic research is required to best handle edge cases such as vessels of the class being in extreme proximity. Finally, this study did not investigate clinical outcome. While our results clearly indicate that surgeons are absorbing anatomical information via the virtual 3D models in a different manner to VRI leading to a distinct change in surgical plan in a fifth of cases, it does not prove that the approach taken with the use of the virtual 3D models is necessarily a better choice in terms of patient outcome. A prospective randomised controlled trial (RCT) examining primary outcomes such as length of stay, complication incidents, blood loss, and OR time will be the ultimate arbiter for healthcare outcomes impact.

## Conclusions

This study demonstrates that the use of interactive virtual 3D models as an adjunct to renal cancer surgery planning influences clinical decisions which can result in changes to surgical approach decisions, as compared to planning with the status quo imaging. Compared to volume-rendered images, virtual 3D models were deemed by urologists to present the patient anatomy in a clearer manner, to increase surgeon confidence in their surgical plan, and to have a higher potential for clinical benefit at MDT, theatre planning, and intra-operative stages.

## Electronic supplementary material

Below is the link to the electronic supplementary material.

Supplementary material 1 (DOCX 877 kb)
