# Supplementary material for: Interactive virtual 3D models of renal cancer patient anatomies alter partial nephrectomy surgical planning decisions and increase surgeon confidence compared to volume-rendered images
Source: Int J Comput Assist Radiol Surg. 2019 Jan 24;14(4):723–32. doi: 10.1007/s11548-019-01913-5 (PMC6420910; doi:10.1007/s11548-019-01913-5)

## Supplementary Figure S1

Static screenshots of the generated, case-specific (A-E along rows), interactive virtual 3D models will the venous/portal venous structure visibility turned off to provide the user with less obstructed view of the relationship of the arterial system to the tumour and excretory structures from an anterior (left) and posterior (right) viewpoint.


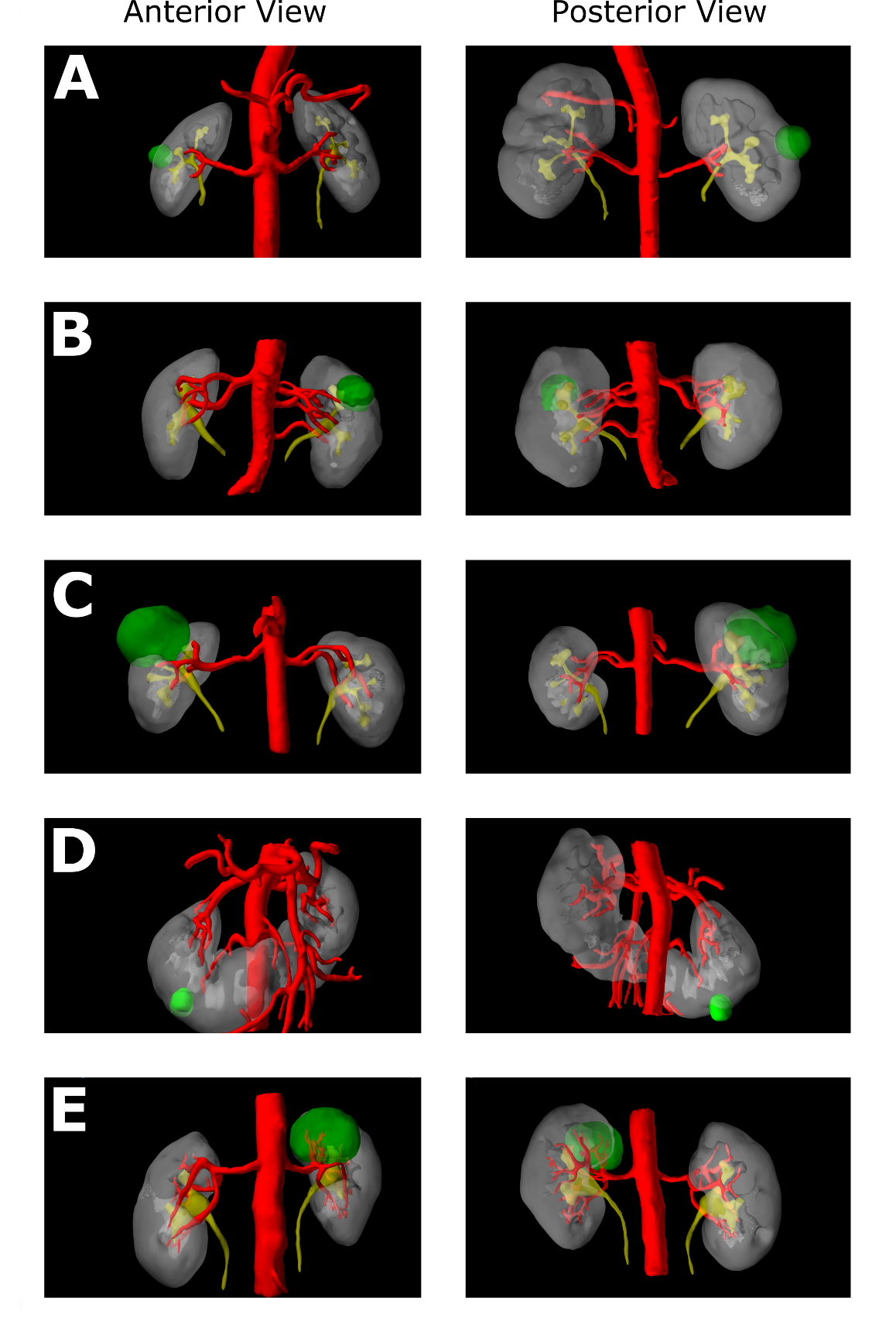

Supplement: Supplementary file 1 — Supplementary material 1 (DOCX 877 kb) [file 11548_2019_1913_MOESM1_ESM.docx]
